# The role of local wisdom ``Ugahari'' and the impact of internet and mobile technology on work-life-balance during COVID-19 outbreak: Data set from malaysian workers

**DOI:** 10.1016/j.dib.2021.107779

**Published:** 2022-01-10

**Authors:** Suhal Kusairi, Suriyani Muhamad, Norizan Abdul Razak, Aji Purba Trapsila

**Affiliations:** a Faculty of Business, Economics and Social Development, Universiti Malaysia Terengganu, Malaysia and Faculty of Economics and Business, Telkom University, Bandung Indonesia; b Faculty of Business, Economics and Social Development, Universiti Malaysia Terengganu, Malaysia; c Faculty of Social Sciences and Humanity, Universiti Kebangsaan Malaysia, Malaysia; d Faculty of Business, Economics and Social Development, Universiti Malaysia Terengganu, Malaysia

**Keywords:** Work-life balance, Flexibility and permeability, Work autonomy, ICT, Local wisdom

## Abstract

This article presents internet and mobile technology (IMT) usage during the pandemic and examines its impact on Malaysian workers' work-life balance. This study also included the Malaysian local value, namely Ugahari, and its role in shaping individual work and personal/family life behavior. The operationalization of variables was developed based on the work-life balance from permeability and flexibility, type of work-life balance consequences, and the theory of planned behavior utilized for local wisdom to characterize the respondents. Data were collected through online surveys and distributed to industries and government agencies in the Urban Area of Malaysia. There are 466 valid and complete questionnaires. The data set has been collected as a reference source for further research regarding the role of local value "Ugahari, especially on work-life balance.

## Specifications Table

**Table:** 

Subject	Social Science
Specific subject area	Social Science
Type of data	Table
How data were acquired	Data is collected through online and offline surveys
Data format	Raw, analyzed, descriptive and statistical data
Parameters for data collection	The data consist of workers in middle management positions and above, located in Kuala Lumpur, Selangor, and Putra Jaya, Malaysia.
Description of data collection	Data collection is done in two ways, namely online and offline. Online data collection was carried out using a monkey survey—the filling period was October 18, 2020, to April 28, 2021. The dataset included 466 valid responses. And the questionnaire is provided as a supplementary file
Data source location	Kuala Lumpur, Selangor and Putra Jaya, Malaysia
Data accessibility	Data is included in this article
Related research article	S. Kusairi, S. Muhammad, N.A. Razak, A.P. Trapsila, Managing Work-Life Balance (WLB) during the COVID-19 Pandemic: The Role of Information and Communication Technology (ICT) and Local Wisdom, Jafeb.

## Value of the Data

• The dataset uses the internet and mobile technology and the role of local wisdom ``Ugahari'' to study their impact on work-life balance.
• This Data is helpful for both public and private policymakers to improve workers' wellbeing during the COVID-19 pandemic.
• The data presented can further discuss the development of work-life balance theory, especially concerning culture and local wisdom.
• Researchers give flexibility to anyone to modify and use in other contexts.

## 1 Data Description

The data in this study is premier data, and it is collected from respondents using the questionnaire. The population frame is workers at the middle level of management from the public and private sectors. Public sectors are divided into clusters based on the department, and private sectors are divided into groups based on the industrial types. The questionnaire is given to all selected respondents. Closed questions were used in this questionnaire, and the options used a Likert scale model. The questionnaire is built on a solid definition as a determinant of how each variable is measured. This process is a critical step of survey research to ensure that the questionnaire items are related to the main problem being studied. The model's evaluation by looking at a loading value of 0.5–0.60 is considered sufficient, and the average variance extracted (AVE) value must be greater than 0.5. Finally, composite reliability by looking at the Cronbach's Alpha value for the condition is > 0.70 [1].

Survey data is broadly divided into two parts: *First*, demographics include gender, age, marital status, race, number of children, and salary (Table 1). Second explorations of all analyzed variables, including the role of Ugahari and the impact of IMT on work-life balance for more detail, can be seen in (Tables 2–4). Furthermore, The dataset, available on the Mendeley Data repository (https://data.mendeley.com/datasets/xnryh937hc/1).

**Table 1:** Respondents' characteristics (*N* = 466).

Characteristics	*N*	%
Demographic characteristics		
Gander		
Male	238	51.07%
Female	228	48.93%
Age		
< 25	71	15.24%
26–30	117	25.11%
31–35	105	22.53%
36–40	71	15.24%
41–45	41	8.80%
46–50	27	5.79%
51–55	24	5.15%
> 55 2	10	2.15%
Marital Status		
Divorce	10	2.15%
Married	260	55.79%
Not married	196	42.06%
Race		
Chinese	93	19.96%
Iban	3	0.64%
Indian	19	4.08%
Kadazan-dusun	2	0.43%
Malay	344	73.82%
Other	5	1.07%
Number of Children		
1	56	12.02%
2	77	16.52%
3	52	11.16%
4	27	5.79%
> 4:	10	2.15%
No children:	244	52.36%
Work-Related Variable		
Wage		
< RM5,000	281	60.30%
RM 5001–RM 7500	80	17.17%
RM 7501–RM 10,000	69	14.81%
RM 10,001–RM 12,500	16	3.43%
RM 12,251–RM 15,000	7	1.50%
> RM 15,000	13	2.79%

**Table 2:** Descriptive results of participants' responses.

Variables		*N*	Min	Max	Mean	Std. Deviation
Permeability						
PE01	I think about my family members when I am at work.	466	1	5	3.59	1.004
PE02	I stop in the middle of my work to address a family concern	466	1	5	3.62	0.969
PE03	I take care of family matters while I am at work.	466	1	5	3.52	0.993
PE04	I receive work-related calls (Phone, Email, WhatsApp, etc.) while I am at home.	466	1	5	3.68	0.973
PE05	I think about work-related concerns while I am at home.	466	1	5	3.37	1.009
PE06	I stop in the middle of my home activities to address a work concern.	466	1	5	3.31	0.978
Flexibility						
FL01	If the need arises, I could leave work early to attend to family-related issues.	466	1	5	3.55	0.939
FL02	I am willing to take time off from work to deal with my family and personal responsibilities.	466	1	5	3.68	0.912
FL03	From a family and personal life standpoint, there is no reason why I cannot rearrange my schedule to meet the demands of my work.	466	1	5	3.49	0.837
FL04	If the need arises, I could work late without affecting my family and personal responsibilities	466	1	5	3.65	0.819
Integration						
In01	I tend to integrate my work and family duties when I work at home	466	1	5	3.35	0.855
In02	I tend to integrate my work and family duties when at work.	466	1	5	3.35	0.838
Autonomy						
AU01	I am allowed to decide how to go about getting my job done (the methods to use).	466	1	5	3.69	0.822
AU02	I can choose the way to go about my job (the procedures to utilize).	466	1	5	3.69	0.813
AU03	I am free to choose the method(s) to use in carrying out my work.	466	1	5	3.64	0.843
AU04	I have control over the scheduling of my work.	466	1	5	3.54	0.869
AU05	I have some control over the sequencing of my work activities (when I do what).	466	1	5	3.57	0.834
Interference						
INT01	The time I spend with my family often causes me not to spend time in activities at work that could be helpful to my career.	466	1	5	2.83	0.971
INT02	When I get home from work, I am often too frazzled to participate in family activities/ responsibilities	466	1	5	2.88	1.006
INT03	I am often so emotionally drained when I get home from work that it prevents me from contributing to my family.	466	1	5	2.93	1.040
INT04	Due to stress at home, I am often preoccupied with family matters at work. (such as children mischief at school/home).	466	1	5	2.71	1.039
INT05	Because I am often stressed from family responsibilities, I have a hard time concentrating on my work.	466	1	5	2.70	1.054
INT06	Tension and anxiety from my family life often weaken my ability to do my job.	466	1	5	2.72	1.089
Segmentation						
SS01	Where I work, people can keep work matters at work.	466	1	5	3.45	0.905
SS02	At my workplace, people are able to prevent work issues from creeping into their home life.	466	1	5	3.21	0.937
SS03	Where I work, people can mentally leave work behind when they go home.	466	1	5	3.23	0.938
IMT						
IC01	How many of the phone calls (cell phone) you make and receive on your phone are personal?	466	1	5	3.39	0.940
IC02	How often do you usually take your mobile phone on a workday to talk to your family or friends?	466	1	5	3.25	0.983
IC03	How many of the phone calls (cell phone) you make and receive on your phone are for work?	466	1	5	3.66	0.930
IC04	How often do you usually answer the mobile for working purposes during a holiday?	466	1	5	3.27	1.017
Ugahari						
UG01	The people around me believe that harmony between life and work is important.	466	1	5	4.06	0.826
UG02	I have the support of people around me to arrange the best time possible so that my responsibilities as a family member and worker can run optimally.	466	1	5	3.95	0.826
UG03	The community around me believes that overwork is one thing that is not good.	466	1	5	3.92	0.861
UG04	I have enough time to interact with friends and build quality relationships with family.	466	1	5	3.83	0.807
UG05	I used to work proportionally.	466	1	5	3.73	0.789
UG06	I limit the additional work to ensure my core task settled well.	466	1	5	3.80	0.804
UG07	I realize that controlling the frequency of technology used, such as mobile technology, email, etc., is important.	466	1	5	3.81	0.880
UG08	People around me realize that excessive use of technology such as mobile phones, the internet etc., can have negative impacts both mentally and physically.	466	1	5	3.65	0.908
UG09	I have the support from the community around me to regulate the use of technology so as not to overdo it.	466	1	5	3.62	0.893

**Table 3:** Measurement model evaluation.

Research construct	The average variance extracted (AVE)	Cronbach's alpha	Composite reliability
Permeability	0.528	0.821	0.87
Flexibility	0.595	0.773	0.854
Integration	0.865	0.844	0.928
Interference	0.655	0.894	0.919
Autonomy	0.686	0.885	0.916
Segmentation	0.752	0.835	0.901
IMT	0.613	0.791	0.613
Ugahari	0.564	0.906	0.921

**Table 4:** Correlations between variables.

	Permeability						Flexibility			
Variable	PE01	PE02	PE03	PE04	PH05	PH06	FL01	FL02	FL03	FL04
Respondent characteristic:										
Gender	−0.076	0.012	0.012	0.000	−0.030	−0.011	−0.022	−0.045	0.005	0.009
Age	−0.076	0.012	−0.014	0.000	−0.030	−0.011	−0.022	−0.045	0.005	0.009
Marital Status	−0.040	−0.103*	−0.124⁎⁎	−0.028	−0.070	−0.048	−0.056	−0.015	−0.062	0.002
Race	−0.095*	−0.104*	−0.082	−0.085	−0.046	−0.029	−0.009	−0.097*	−0.149⁎⁎	−0.061
Number of Chidren	0.029	0.060	.123⁎⁎	0.076	0.088	.094*	−0.008	−0.027	0.061	0.042
Wage	0.078	.109*	.115*	.113*	.213⁎⁎	.201⁎⁎	.146⁎⁎	.134⁎⁎	.139⁎⁎	.146⁎⁎
Integration										
IN01	.125⁎⁎	0.087	.195⁎⁎	.265⁎⁎	.301⁎⁎	.306⁎⁎	.147⁎⁎	.220⁎⁎	.285⁎⁎	.375⁎⁎
IN02	.197⁎⁎	.167⁎⁎	.294⁎⁎	.185⁎⁎	.202⁎⁎	.218⁎⁎	.183⁎⁎	.204⁎⁎	.250⁎⁎	.363⁎⁎
Autonomy										
AU01	.125⁎⁎	0.087	.195⁎⁎	.265⁎⁎	.301⁎⁎	.306⁎⁎	.147⁎⁎	.220⁎⁎	.285⁎⁎	.375⁎⁎
AU02	.197⁎⁎	.167⁎⁎	.294⁎⁎	.185⁎⁎	.202⁎⁎	.218⁎⁎	.183⁎⁎	.204⁎⁎	.250⁎⁎	.363⁎⁎
AU03	.200⁎⁎	.169⁎⁎	.171⁎⁎	.216⁎⁎	.260⁎⁎	.253⁎⁎	.295⁎⁎	.305⁎⁎	.291⁎⁎	.341⁎⁎
AU04	.171⁎⁎	.150⁎⁎	.175⁎⁎	.247⁎⁎	.255⁎⁎	.213⁎⁎	.327⁎⁎	.270⁎⁎	.281⁎⁎	.290⁎⁎
AU05	.217⁎⁎	.245⁎⁎	.262⁎⁎	.292⁎⁎	.325⁎⁎	.267⁎⁎	.284⁎⁎	.318⁎⁎	.307⁎⁎	.303⁎⁎
Interference										
INT01	0.002	−0.020	.117*	−0.054	−0.016	0.021	−0.009	−0.085	0.022	−0.007
INT02	−0.029	−0.030	0.006	−0.029	0.030	0.030	0.033	0.051	0.038	0.010
INT03	−0.021	−0.028	0.040	−0.011	0.077	0.040	0.032	−0.003	0.041	0.055
INT04	0.080	0.068	.155⁎⁎	−0.019	0.083	.115*	0.065	0.011	.129⁎⁎	.100*
INT05	0.050	0.060	.152⁎⁎	−0.035	0.086	0.090	0.072	0.020	.109*	.113*
INT06	0.053	0.047	.114*	−0.028	.098*	.116*	0.034	−0.009	0.043	0.045
Segmentation										
SS01	.161⁎⁎	.117*	.174⁎⁎	0.066	.102*	0.090	.149⁎⁎	.178⁎⁎	0.067	0.082
SS02	.164⁎⁎	0.072	.130⁎⁎	−0.012	−0.005	0.022	.176⁎⁎	.194⁎⁎	0.057	0.058
SS03	.108*	0.044	.112*	0.008	0.006	0.035	.119⁎⁎	.096*	0.064	.106*
IMT										
IC01	.190⁎⁎	.183⁎⁎	.235⁎⁎	.195⁎⁎	.214⁎⁎	.184⁎⁎	.148⁎⁎	.112*	.121⁎⁎	0.079
IC02	.214⁎⁎	.197⁎⁎	.326⁎⁎	.158⁎⁎	.207⁎⁎	.180⁎⁎	.156⁎⁎	.178⁎⁎	.238⁎⁎	.123⁎⁎
IC03	.146⁎⁎	.113*	.149⁎⁎	.284⁎⁎	.284⁎⁎	.204⁎⁎	.163⁎⁎	.181⁎⁎	.244⁎⁎	.209⁎⁎
IC04	.132⁎⁎	.196⁎⁎	.236⁎⁎	.265⁎⁎	.393⁎⁎	.345⁎⁎	.192⁎⁎	.149⁎⁎	.220⁎⁎	.256⁎⁎
Ugahari										
UG01	.169⁎⁎	.192⁎⁎	.179⁎⁎	.283⁎⁎	.233⁎⁎	.166⁎⁎	.169⁎⁎	.200⁎⁎	.248⁎⁎	.300⁎⁎
UG02	.182⁎⁎	.156⁎⁎	.190⁎⁎	.237⁎⁎	.179⁎⁎	.170⁎⁎	.176⁎⁎	.193⁎⁎	.224⁎⁎	.234⁎⁎
UG03	.243⁎⁎	.219⁎⁎	.201⁎⁎	.165⁎⁎	.171⁎⁎	.112*	.198⁎⁎	.165⁎⁎	.283⁎⁎	.329⁎⁎
UG04	.175⁎⁎	.137⁎⁎	.148⁎⁎	.163⁎⁎	.150⁎⁎	.151⁎⁎	.132⁎⁎	.147⁎⁎	.189⁎⁎	.250⁎⁎
UG05	.192⁎⁎	.133⁎⁎	.158⁎⁎	.133⁎⁎	.205⁎⁎	.132⁎⁎	.197⁎⁎	.204⁎⁎	.178⁎⁎	.190⁎⁎
UG06	.176⁎⁎	.179⁎⁎	.181⁎⁎	.153⁎⁎	.177⁎⁎	.181⁎⁎	.144⁎⁎	.198⁎⁎	.194⁎⁎	.171⁎⁎
UG07	0.09	0.078	0.071	.126⁎⁎	0.082	0.072	.151⁎⁎	.190⁎⁎	.171⁎⁎	.168⁎⁎
UG08	.200⁎⁎	.168⁎⁎	.237⁎⁎	.124⁎⁎	.181⁎⁎	.170⁎⁎	.246⁎⁎	.185⁎⁎	.280⁎⁎	.212⁎⁎
UG09	.192⁎⁎	.139⁎⁎	.235⁎⁎	.135⁎⁎	.159⁎⁎	.144⁎⁎	.138⁎⁎	.151⁎⁎	.202⁎⁎	.196⁎⁎

⁎⁎ Correlation is significant at the 0.01 level (2-tailed).

⁎ Correlation is significant at the 0.05 level (2-tailed).

## 2 Experimental Design, Materials and Methods

The questionnaires were distributed in two ways: first, using the traditional method (offline) by visiting the cluster of sampled companies. They were secondly using online survey through https://www.surveymonkey.com/r/5YGVBCY. In the process, most of the respondents came from Kuala Lumpur and Selangor. Respondents from Putra Jaya Malaysia are devoted to respondents who work in the public sector. The average time for filling out the questionnaire per respondent is 30 min. In general, the number of respondents who filled in was 580, but 466 respondents met the requirements to continue the analysis after the screening—the filling period from October 18, 2020, to April 28, 2021.

Conclusive empirical findings from previous research will support the development of research instruments. The development of the model in this study consists of two exogenous variables, namely the internet and mobile technology (IMT) [2] and Malaysian local wisdom "Ugahari." For variables endogenous there are eight constructs namely: Integration [3], Autonomy [4], Interference [5], Segmentation [6], Job satisfaction [7], job stress [8], permeability [9] and flexibility [3]. Most of the measurements in this study use a five-point Likert scale where 1 = Strongly Disagree, 3 = Neither Agree nor Disagree, and 5 = Strongly Agree. The rating scales allow results to be analyzed and interpreted statistically.

The data screening process is carried out in two ways: First, screening the completeness of filling out all the questions in the questionnaire. At this stage, the researcher ensures that all the data collected is complete and suitable for analysis. Second, check whether there are irregularities in filling out; for example, respondents' names are not familiar, industries not in the Google database, and others; this is important to ensure the quality of the data obtained.

## Ethics Statement

All procedures performed in studies followed the ethical standard of University Malaysia Terengganu. Informed consent was obtained from all respondents involved in the study. Participants were informed that the survey was anonymous.

## Data Availity

The Role Of Local Wisdom ``Ugahari'' And The Impact Of Internet And Mobile Technology On Work-Life-Balance During COVID-19 Outbreak: Data Set From Malaysian Workers (original Data) (mendelay Data).

## CRediT authorship contribution statement

**Suhal Kusairi:** Conceptualization, Methodology. **Suriyani Muhamad:** . **Norizan Abdul Razak:** . **Aji Purba Trapsila:** Data curation, Writing – original draft.

## Declaration of Competing Interest

The authors state that they have no competing interests and have no conflicts of interest concerning the research described in this paper.

## Appendix Supplementary materials

Image, application 1

Image, application 2
