# Supplementary material for: The role of local wisdom ``Ugahari'' and the impact of internet and mobile technology on work-life-balance during COVID-19 outbreak: Data set from malaysian workers
Source: Data Brief. 2022 Jan 10;40:107779. doi: 10.1016/j.dib.2021.107779 (PMC8743796; doi:10.1016/j.dib.2021.107779)
Supplement: Supplementary file 2 [file mmc2.docx]

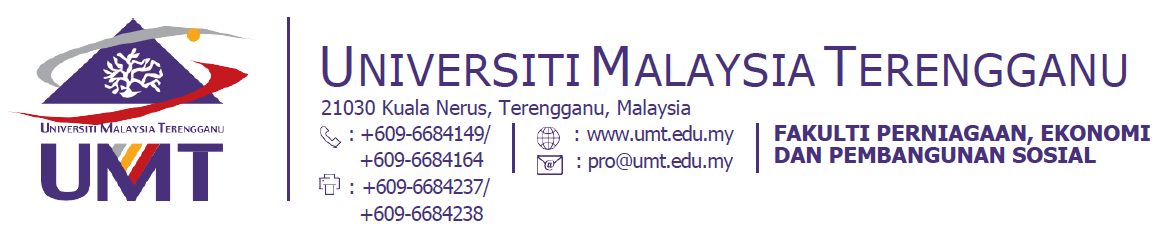


Dear Respondent,

Ladies and Gentlemen, allow us Prof. Madya Dr. Suhal Kusairi, Prof. Madya Dr. Suriyani Muhamad, Prof. Datin Dr. Norizan Abdul Razak and Aji Purba Trapsila, SEI., MEI. We are currently conducting research under the title " Industrial Revolution 4.0 and Work-Life-Balance: Towards a Theoretical Model of Digital Technology Use with Ugahari Concept". The terms Ugahari in this research refers to “Moderate, Intermediate and Simple”. Additionally this study was funded by the Fundamental Research Grant Scheme (FRGS) - the Minister of Higher Education of Malaysia. The intended respondents in this research are middle management and above.

This filling takes about 30 minutes to complete. All data and information obtained from this research process are confidential, and are used entirely for academic purposes. The study will focus mainly on the following objectives:

- 1. Evaluating the Ugahari value, level of internet usage and mobile technology and work-life balance and how it will contribute to the betterment of society.
  2. Developing the model Ugahari and level of internet usage and mobile technology to explain the WLB of workers.
  3. Examining the influence of Ugahari values and the level of internet usage and mobile technology toward the WLB of workers.

All the data gathered will be kept confidential and used only for research purpose. It will not be used in a manner which would allow identification of your individual responses. Participation is strictly voluntary and you may refuse to participate at any time. Completion and return of the survey form will indicate your willingness to participate in this study.

Thank you very much for your willingness to fill out this questionnaire. And hopefully this research can provide benefits for the development of Work-life balance in Malaysia study. If you require additional information or have questions, please contact us via email indicated below.

Sincerely,

Prof. Madya Dr. Suhal Kusairi

Lecturer of Universiti Malaysia Terengganu

E-mail: [suhal@umt.edu.my](mailto:suhal@umt.edu.my)

**PART 1: PERSONAL DEMOGRAPHIC**

- 1. Gender: 🖵 Male 🖵 Female
  2. Religion: 🖵 Islam 🖵 Catholic 🖵 Buddha 🖵 Christian 🖵 Hindu

🖵 Other (please specify); __________________

- 1. Age: 🖵 <25 🖵 26 – 30 🖵 31 – 35 🖵 36 – 40 🖵 41 – 45 🖵 46 – 50 🖵 51 – 55

🖵 >55

- 1. Marital status: 🖵 Married 🖵 not married 🖵 Divorce
  2. Race: 🖵 Malay 🖵 Chinese 🖵 Indian 🖵 Iban 🖵 Kadazan-dusun

🖵 Other (please specify); __________________

- 1. Level of educational:

🖵 SPM/STPM 🖵 Diploma/Bachelor’s Degree 🖵 PhD Degree

🖵 Master’s Degree 🖵 Professional Certificate

🖵 Other (please specify); __________________

- 1. Job position (public sector): 🖵 Deputy Secretary 🖵 Senior Under-Secretary (Management)

🖵 Department in Chief

🖵 Other (please specify); __________________

- 1. Job position (private sector): 🖵 Executive 🖵 Manager of Department 🖵 Top Management

🖵 Other (please specify); __________________

- 1. Length of employment:

🖵 < 3 years 🖵 4 – 9 years 🖵 10 – 15 years

🖵 16 – 21 years 🖵 22 – 27 years 🖵 28 – 33 years 🖵 > 33 years

- 1. Number of salary:

🖵 < RM5,000 🖵 RM 7,501 – RM 10,000 🖵 RM 12,251 – RM 15,000

🖵 RM 5001 – RM 7500 🖵 RM 10,001 – RM 12,500 🖵 > RM 15,000

- 1. Number of children

🖵 No Children 🖵 2 🖵 4

🖵 1 🖵 3 🖵 > 4

**PART 2: COMPANY / ORGANIZATION INFORMATION /**

- 1. Government organization (if related):

🖵Ministry: ______________________ 🖵 Department / Director: ________________________

- 1. Business organization (if related)

Name of the company : ____________________________________

Industrial Sector : ____________________________________

Departments : ____________________________________

- 1. Year of establishment:

🖵 < 5 years 🖵 6 – 9 years 🖵 10 – 13 years

🖵 14 – 17 years 🖵 18 – 21 years 🖵 > 21 years

- 1. Number of employees:

🖵 < 31 🖵 31 – 75 🖵 76 – 200 🖵 200 – 500 🖵 501 – 1000 🖵 > 1000

**PART 3: WORK-LIFE DIMENSION**

Please indicate the extent to which you agree or disagree about the ***Work-Life Dimension*** by putting a tick mark (🗸) in the appropriate box using the scale below:

| ***Strongly Disagree (1)*** | ***Disagree (2)*** | ***Neutral (3)*** | ***Agree (4)*** | ***Strongly Agree (5)*** |
| --- | --- | --- | --- | --- |

**Permeability**

|  | | **1** | **2** | **3** | **4** | **5** |
| --- | --- | --- | --- | --- | --- | --- |
| PE01 | I think about my family members when I am at work. |  |  |  |  |  |
| PE02 | I stop in the middle of my work to address a family concern. |  |  |  |  |  |
| PE03 | I take care of family matters while I am at work. |  |  |  |  |  |
| PE04 | I receive work-related calls (phone, email, whatsapp, etc) while I am at home. |  |  |  |  |  |
| PE05 | I think about work-related concerns while I am at home. |  |  |  |  |  |
| PE06 | I stop in the middle of my home activities to address a work concern. |  |  |  |  |  |

**Flexibility**

|  | | **1** | **2** | **3** | **4** | **5** |
| --- | --- | --- | --- | --- | --- | --- |
| FL01 | If the need arises, I could leave work early to attend to family-related issues. |  |  |  |  |  |
| FL02 | I am willing to take time off from work to deal with my family and personal responsibilities. |  |  |  |  |  |
| FL03 | From a family and personal life standpoint, there is no reason why I cannot rearrange my schedule to meet the demands of my work. |  |  |  |  |  |
| FL04 | If the need arises, I could work late without affecting my family and personal responsibilities. |  |  |  |  |  |

**PART 4: WORK-LIFE TYPES**

Please indicate the extent to which you agree or disagree about the ***Work-Life Types*** by putting a tick mark (🗸) in the appropriate box using the scale below:

| ***Strongly Disagree (1)*** | ***Disagree (2)*** | ***Neutral (3)*** | ***Agree (4)*** | ***Strongly Agree (5)*** |
| --- | --- | --- | --- | --- |

**Integration**

|  | | **1** | **2** | **3** | **4** | **5** |
| --- | --- | --- | --- | --- | --- | --- |
| **In39** | I tend to integrate my work and family duties when I work at home. |  |  |  |  |  |
| **In40** | I tend to integrate my work and family duties when at work. |  |  |  |  |  |

**Autonomy**

| **Work Method Autonomy** | | **1** | **2** | **3** | **4** | **5** |
| --- | --- | --- | --- | --- | --- | --- |
| AU01 | I am allowed to decide how to go about getting my job done (the methods to use). |  |  |  |  |  |
| AU02 | I can choose the way to go about my job (the procedures to utilise). |  |  |  |  |  |
| AU03 | I am free to choose the method(s) to use in carrying out my work. |  |  |  |  |  |
| AU04 | I have control over the scheduling of my work. |  |  |  |  |  |
| AU05 | I have some control over the sequencing of my work activities (when I do what). |  |  |  |  |  |

**Interference**

| **Time-based work interference with family** | | **1** | **2** | **3** | **4** | **5** |
| --- | --- | --- | --- | --- | --- | --- |
| INT01 | The time I spend with my family often causes me not to spend time in activities at work that could be helpful to my career. |  |  |  |  |  |
| INT02 | When I get home from work I am often too frazzled to participate in family activities/ responsibilities. |  |  |  |  |  |
| INT03 | I am often so emotionally drained when I get home from work that it prevents me from contributing to my family. |  |  |  |  |  |
| INT04 | Due to stress at home, I am often preoccupied with family matters at work. (such as children mischief at school/home, etc.). |  |  |  |  |  |
| INT05 | Because I am often stressed from family responsibilities, I have a hard time concentrating on my work. |  |  |  |  |  |
| INT06 | Tension and anxiety from my family life often weakens my ability to do my job. |  |  |  |  |  |

**Segmentation**

|  | | **1** | **2** | **3** | **4** | **5** |
| --- | --- | --- | --- | --- | --- | --- |
| SS01 | Where I work, people can keep work matters at work. |  |  |  |  |  |
| SS02 | At my workplace, people are able to prevent work issues from creeping into their home life. |  |  |  |  |  |
| SS03 | Where I work, people can mentally leave work behind when they go home. |  |  |  |  |  |

**PART 5: WORK-LIFE CONSEQUENCES**

Please indicate the extent to which your opinion about the **Work-Life Consequences** by putting a tick mark (🗸) in the appropriate box using the scale below:

| ***Strongly Disagree (1)*** | ***Disagree (2)*** | ***Neutral (3)*** | ***Agree (4)*** | ***Strongly Agree (5)*** |
| --- | --- | --- | --- | --- |

**Job Satisfaction**

| **Job Satisfaction** | | **1** | **2** | **3** | **4** | **5** |
| --- | --- | --- | --- | --- | --- | --- |
| JS01 | Most days I am enthusiastic about my work. |  |  |  |  |  |
| JS02 | I find real enjoyment in my work. |  |  |  |  |  |
| JS03 | The work I do on my job is meaningful to me. |  |  |  |  |  |
| JS04 | All in all, I am satisfied with my job. |  |  |  |  |  |

**Job Stress**

**PART 6: USE OF THE INTERNET AND MOBILE TECHNOLOGY**

| **Job Stress** | | **1** | **2** | **3** | **4** | **5** |
| --- | --- | --- | --- | --- | --- | --- |
| JSt01 | I work under a great deal of tension. |  |  |  |  |  |
| JSt02 | My working environment is very stressful. |  |  |  |  |  |
| JSt03 | It makes me tense to think about my job. |  |  |  |  |  |
| JSt04 | While at work, I feel there is too much pressure to get things done. |  |  |  |  |  |
| JSt05 | I have unwanted stress as a result of my present job. |  |  |  |  |  |
| JSt06 | I feel “burned-out” after a full day of work. |  |  |  |  |  |

Please indicate the extent to which you never or almost always about the ***Use of the internet and mobile technology*** by putting a tick mark (🗸) in the appropriate box using the scale below:

| ***Never (1)*** | ***Seldom (2)*** | ***Occasionally (3)*** | ***To a Considerable Degree (4)*** | ***Almost Always (5)*** |
| --- | --- | --- | --- | --- |

**Frequency of Use**

|  | | **1** | **2** | **3** | **4** | **5** |
| --- | --- | --- | --- | --- | --- | --- |
| IC01 | How many of the phone calls (cell phone) you make and receive on your phone are personal? |  |  |  |  |  |
| IC02 | How often do you usually take your mobile phone on work-day to talk to your family or friends? |  |  |  |  |  |
| IC03 | How many of the phone calls (cell phone) you make and receive on your phone are for work? |  |  |  |  |  |
| IC04 | How often do you usually answer the mobile for working purposes during holiday ? |  |  |  |  |  |

Please indicate the extent to which you agree or disagree about the ***Ugahari*** by putting a tick mark (🗸) in the appropriate box using the scale below:

**PART 7: UGAHARI values: moderate, intermediate and simple**

| ***Strongly Disagree (1)*** | ***Disagree (2)*** | ***Neutral (3)*** | ***Agree (4)*** | ***Strongly Agree (5)*** |
| --- | --- | --- | --- | --- |

**Ugahari behavior on work**

| **Subjective norms** | | **1** | **2** | **3** | **4** | **5** |
| --- | --- | --- | --- | --- | --- | --- |
| UG01 | The people around me believe that harmony between life and work is important. |  |  |  |  |  |
| UG02 | I have the support of people around me to arrange the best time possible so that my responsibilities as a family member and worker can run optimally. |  |  |  |  |  |
| UG03 | The community around me believes that overwork is one thing that is not good. |  |  |  |  |  |
| UG04 | I have enough time to interact with friends and build quality relationships with family. |  |  |  |  |  |
| UG05 | I used to work proportionally. |  |  |  |  |  |
| UG06 | I limit the additional work to ensure my core task settled well. |  |  |  |  |  |
| UG07 | I realise that controlling the frequency of technology used such as mobile technology, email, etc. is important. |  |  |  |  |  |
| UG08 | People around me realize that excessive use of technology such as mobile phone, internet etc can have negative impacts both mentally and physically. |  |  |  |  |  |
| UG09 | I have the support from the community around me to regulate the use of technology so as not to overdo it. |  |  |  |  |  |

- 1. Do you have any suggestion/comments?

***End of Questionnaire***

***THANK YOU***
